# Let’s Read a Poem! What Type of Poetry Boosts Creativity?

**DOI:** 10.3389/fpsyg.2018.01781

**Published:** 2018-09-21

**Authors:** Małgorzata Osowiecka, Alina Kolańczyk

**Affiliations:** ^1^Warsaw Faculty of Psychology, SWPS University of Social Sciences and Humanities, Warsaw, Poland; ^2^Faculty in Sopot, SWPS University of Social Sciences and Humanities, Sopot, Poland

**Keywords:** creativity, divergent thinking, metaphor, poetry reception, language

## Abstract

Poetry is one of the most creative uses of language. Yet the influence of poetry on creativity has received little attention. The present research aimed to determine how the reception of different types of poetry affect creativity levels. In two experimental studies, participants were assigned to two conditions: poetry reading and non-poetic text reading. Participants read poems (Study 1 = narrative/open metaphors; Study 2 = descriptive/conventional metaphors) or control pieces of non-poetic text. Before and after the reading manipulation, participants were given a test to determine levels of divergent thinking (DT; i.e., fluency, flexibility, and originality). Additionally, in both studies, the impact of frequent contact with poetry was examined. In Study 1 (*N* = 107), participants showed increased fluency and flexibility after reading a narrative poem, while participants who read the non-poetic text showed a decrease in fluency and originality. In Study 2 (*N* = 131) reception of conventional, closed metaphorization significantly lowered fluency and flexibility of thinking (compared to reading non-poetic text). The most critical finding was that poetry exposure could either increase or decrease creativity level depending on the type of poetic metaphors and style of poetic narration. Furthermore, results indicate that long-term exposure to poetry is associated with creativity. This interest in poetry can be explained by an ability to immerse oneself in a poetry content (i.e., a type of empathy) and the need for cognitive stimulation. Thus, this paper contributes a new perspective on exposure to poetry in the context of creativity and discusses possible individual differences that may affect how this type of art is received. However, future research is necessary to examine these associations further.

## Introduction

Creativity is often understood in different ways. In an elitist view, creativity means eminent works of art created by great, gifted artists. In contrast, creativity has also been described as a common cognitive process, which can be improved (Finke et al., 1992). This more popular approach has been labeled by Csikszentmihalyi (1996) as “little c Creativity." Previous research (Mednick, 1962) has shown that creative thinking is based on flatter concept hierarchies, enabling remote associations to be more easily made. Csikszentmihalyi states that this kind of creativity is part of everyday human life, and can be observed even in young children. This type of “common" creativity results in more efficient problem solving, better performance on tasks measuring creative potential, and can even bring about the production of outstanding works of art. The current research concentrates on “little c Creativity," which can be improved by specific interventions under specific circumstances, and then observed and measured (Guilford, 1950; Finke et al., 1992; Runco, 1999).

In this article, we examined whether the creative potential of a poem can be beneficial for receivers by testing whether one-time reception of poetry can influence the quality of divergent thinking (DT; i.e., multidirectional and/or potentially creative thinking). Additionally, we investigated if this impact depends on the type of poetic metaphors and/or the style of poetic narration.

There are several studies that have examined how humans produce metaphors (Paivio, 1979; Chiappe and Chiappe, 2007; Silvia and Beaty, 2012; Beaty and Silvia, 2013), but little is known about metaphor comprehension, especially within the context of poetry. This research has inspired many books that attempt to teach the skills necessary to generate imaginative and interesting metaphors (e.g., Plotnik, 2007). It may be that the ability to associate remote ideas, facts, and elements of the environment, which is a key factor in metaphor production, may also be a key factor in creativity. Thus, these skills that can be taught to improve metaphorization may also overlap with skills to improve general creative ability.

Most psychological research on poetry has focused on the influence of text structure (i.e., rhythm, rhymes) on emotional reception of poems (e.g., Jakobson, 1960; Turner and Pöppel, 1983; Lerdahl, 2001; Obermeier et al., 2013). Additionally, many studies that have focused on poets’ creativity have also collected data revealing links between mental disorders and functioning (e.g., Stirman and Pennebaker, 2001; Djikic et al., 2006). Further, previous research has also examined the relationship between poetic training and creativity (e.g., Baer, 1996; Andonovska-Trajkovska, 2008; Cheng et al., 2010). However, the current manuscript focuses on the influence of poems as creative products that may affect receivers’ levels of creative thinking. This influence, however, likely depends on the type of poetry received.

The efficiency of DT is a key measure of idea generation (e.g., Baer, 1996; Runco, 1999; Nęcka, 2012). In contrast to convergent thinking, DT enables problem solving in diverse and potentially valuable ways. It often involves redefining the problem, referring to analogies, redirecting one’s thoughts, and breaking barriers in thinking. Previous research has found that spreading activation in the semantic network is indicative of DT (Martindale, 1989; Ashton-James and Chartrand, 2009; Kaufman and Beghetto, 2009). Developing associations between distant ideas is a basic mechanism of creative thinking (Mednick, 1962). For instance, Benedek et al. (2012) provided evidence that the ability to generate remote associations makes creative problem solving easier. Gilhooly et al. (2007) showed that ignoring close associations (but choosing remote ones) and breaking the stiff, typical relationships between ideas plays a crucial role in effective DT. The current studies are based on the hypothesis that the process of DT can be supported by poetry comprehension.

Poetry, which contains remote associations described through metaphors and analogies, combines non-related notions in atypical ways (Lakoff and Johnson, 2003). In general, metaphoric expression often involves mapping between abstract and more concrete concepts (Glucksberg, 2001, 2003); therefore, the comprehension of metaphors requires the activation of a broader set of semantic associations. This is due to connecting two remote parts of a metaphor (theme and vehicle) into a meaningful expression (Paivio, 1979; Kenett et al., 2018). Poetry reception can involve readiness to notice similarities between remote categories, which can be a crucial ability in generating creative ideas (e.g., Mednick, 1962; Koestler, 1964; Martindale, 1989). Training in metaphorical thinking results in the broadening of categories (Nęcka and Kubiak, 1989), which leads to increased DT (Trzebiński, 1981). Glucksberg et al. (1982) have shown that poetry reading broadens the scope of associations. Metaphor, based on remote associations, provides a new way of understanding reality and human feelings. In addition to fostering multidirectional and creative thinking, metaphor can also help individuals adjust to the surrounding world (Kolańczyk, 1991; Nęcka, 2012). Metaphorization is, structurally, the most essential element of the poetic art (e.g., Lakoff and Johnson, 2003; Kovecses, 2010). Rhythm, syllabification, and word combinations in well-written poetry construct a meaningful whole aside from very remote notions (Csikszentmihalyi, 1996). Thus, poetry comprehension can change readers’ DT; however, this impact likely depends on type of poetic metaphors and the narration used by the poet.

Thinking expressed in metaphors always involves the flexible activation and manipulation of acquired knowledge (Benedek et al., 2014); even though metaphors are not always creative, even in poetry. Understanding a conventional metaphor is not intellectually challenging: comprehending such expressions is based on the retrieval of well-known meaning from memory (Kenett et al., 2018). For example, love can be understood metaphorically as a nutrient. The metaphors “starved for affection” and “given strength by love” are not particularly creative, as they are based on a highly conventional metaphor (i.e., love = nutrient). These metaphors are ostensibly viewed as new by receivers of poetry, although they are not flexible or original. Hausman (1989) writes about two specific types of metaphors; one he describes as impoverished, frozen, and closed; the other, he refers to as original, divergent, and open. It seems logical to use terms like closed/convergent and open/divergent when referring to metaphors, which can emphasize a functional dimension of how these types of metaphors are used in poetry and casual language. To the best of our knowledge, however, previous research has never introduced this distinction in terms of differences between metaphors. Instead, Beaty and Silvia (2013) uses the metaphor labels *conventional* (i.e., familiar) and *creative* (i.e., novel).

Until now, no typologies of metaphors have been introduced that highlight differences in how poetry is constructed and how this impacts recipients. It seems that poetry uses at least these two kinds of metaphorization. Both of these can be adaptive for the recipient, because creativity requires both accommodation and assimilation (Ayman-Nolley, 2010). Therefore, recipients’ reception of novel and open metaphors could result in more flexible and original thinking, whereas reception of conventional, well known, and closed metaphors could result in less flexible and less creative problem-solving.

In addition to the types of metaphors used, poetry is also characterized by content. One conceptualization of poetry describes it as a certain type of story, which is a separate and coherent whole, through which people express their thoughts and/or opinions (Heiden, 2014). In this case, the author can bring an abstract idea closer to the reader through narrative imagery. This type of poetry can result in the receiver taking on another’s (i.e., the author’s) point of view, hence improving creativity. Moreover, this narrative type of poetry is an open task for readers, because understanding is reached based on the receiver’s own experience and understanding. The second type, noncreative poetry, is more conservative, and includes variously structured, commonplace (i.e., conventional) metaphors, which are often clichés based on common-sense regularities, and are sometimes the contents of parables or prayers. Metaphors in this type of poetry delineate and conventionalize meaning; they describe the world in ways known to everyone (e.g., Lakoff and Turner, 1989; Gibbs, 1994; Lakoff and Johnson, 2003; Kovecses, 2010).

The general goals of this research were to determine whether the reception of poetry stimulates creative thinking, and whether poetry’s impact on creativity varies depending on the type of poetry. Accordingly, we formulated the following research hypotheses:

1. Reception of an unconventional, open metaphor poem will stimulate the generation of creative ideas (i.e., improves DT from baseline).
2. Reception of conventional poetry either will not influence, or will negatively influence the generation of creative ideas (i.e., no increase or decrease in DT from baseline).
3. DT will be increased after the reception of open metaphor poetry, when compared to reading a neutral text.
4. DT will be decreased after reception of conventional poetry, when compared to a neutral text.

In Study 1, participants were exposed to a poem with narrative imagery expressing an author’s point of view and utilizing open metaphors. In Study 2, participants were exposed to a conventional poem that employed a biographical approach, comprised of commonplace metaphors and aphorisms.

## Study 1

### Methods

#### Participants

Participants were recruited from high-school classes. All participants resided in Poland. A total of 107 participants completed the study (*M* age = 17.46; *SD* = 1.03; 53 female). Students from the pool were randomly assigned to one of two groups. Upon entering the lab, participants were given a consent form and a brief explanation of the study procedures. The study was conducted in a group setting, with the number of participants ranging from 10 to 15. Participants provided written, informed consent, and were free to withdraw from the research at any time without giving reason or justification for withdrawing. Minors participated in research with written parental consent. Participants received points for behavior as compensation. Their participation was anonymous. The study was approved by a local ethics committee (clearance number: WKE/S 15/VI/1).

### Materials

#### DT Measurement

To measure DT, participants were administered versions of the Question Generation task (Chybicka, 2001). This task was conducted using a test-retest design (to observe creativity change). Participants listed as many questions as they could regarding an unambiguous picture (baseline image from Chybicka, 2001; post-test, a comparable version from Corbalan and Lopez, 1992). The fluency, flexibility, and the originality of answers were evaluated by three independent judges. Fluency was the total number of meaningful responses given by participant; flexibility (i.e., diversity of categories) was measured as the number of different categories; and originality was calculated as the number of original, novel, and interesting responses.

#### Poetry—Szymborska’s Poem

In Study 1, we chose Szymborska (2012) poem *Utopia* as an example of narrative, non-rhythmic poetry. In *Utopia*, Szymborska creates a sort of plot or story, which she conveys to the reader in a very metaphorical, condensed form. Szymborska’s narration in Utopia is characterized by ethical and metaphysical themes (e.g., “*As if all you can do here is leave and plunge, never to return, into the depths. Into unfathomable life & The Tree of Understanding, dazzlingly straight and simple, sprouts by the spring called Now I Get It”*). Six independent judges, all of which were Polish language teachers, filled in a short scale which contained three questions about affectivity of the chosen poem (e.g., “*the poem is neutral*”). They confirmed that the poem was emotionally stable, allowing for control over the influence of both rhythm and emotion on participants’ creativity.

#### Control Text

For the control text, we used the description of a cooking device (Speedcook, RPOL, Mielec, Poland). This description approximated the word count of a poem and did not contain any metaphors (e.g., “*Our kitchen appliance has a classic, elegant design. This device could replace every cooking appliance*, *a steam cooking tool, and a juicer”*). Device descriptions are often made according to the same pattern and in a comparable way. The description that we used contained close, functional associations between concepts. The text is constructed to provide concrete information to the recipient. The device description was obtained from an Internet website (Wachowicz, 2014).

#### Contact With Poetry Scale

We developed a scale to measure poetry contact that addressed passion, as well as frequency of reading poetry and taking part in poetic meetings. Agreement/disagreement with statements was assessed. Statements included “*I am passionate about poetry,”* “*In my free time, I very often read poems,”* “*I write poems and share my work with others,” “I have several favorite poets,” “Sometimes, I put down my creative thoughts onto paper,” and “I was once an unpublished writer.”* Participants answered the five items on a 5-point scale from 1 = strongly disagree to 5 = strongly agree. The reliability of the tool, as measured by internal consistency, was satisfactory (Cronbach’s α = 0.83).

### Procedure

First, participants read introductory information highlighting the importance of their participation in the study and a confidentiality statement (assuring that participants would remain anonymous and encouraging them to answer all questions truthfully). Then, participants received the first version of the Question Generation Task (Chybicka, 2001). Participants wrote questions about a picture printed on a piece of paper for 10 min. Next, participants were randomized into one of two groups: (a) the experimental group, which read the poem; or (b) the control group, which read the cooker description. Participants were instructed to silently read the poem twice, in a calm and attentive manner (Kraxenberger and Menninghaus, 2016). After reading the text, participants answered two questions; one regarding understanding the content (“*I understand the meaning of the text”)* and the other an affective estimation of the text (“*In my opinion, the text is pleasant*”). Items were rated on a 6-point scale, with response options ranging from 1 (*strongly disagree*) to 5 (*strongly agree*). Then, participants completed a parallel version of the drawing from the Question Generation Task (Corbalan and Lopez, 1992). Finally, participants completed the devised scale concerning contact with poetry. Duration of the entire procedure was approximately 35 min. After completing the scale, participants were debriefed and thanked for their participation. We also collected postal addresses from participants who were interested in the results.

Data were analyzed using SPSS 24 (IBM, Armonk, NY, United States). The data from all participants were included in analyses and a significance level of *p* < 0.05 was adopted for all tests.

### Results

All three DT indicators were scored by three independent raters. A Kendall’s *W* of 1.00 was calculated for fluency at both time points; a *W* of 0.75 and 0.72 for flexibility in the first and the second measurement, respectively; and 0.76 for originality in both measurements (*W* greater than 0.70 = good concordance). All indicators were analyzed separately via three repeated-measures analyses of variances (ANOVAs) with effect of measurement (first vs. second) as the within-subjects factor and group (poetry vs. description) as the between-subjects factor.

**FIGURE 1:**
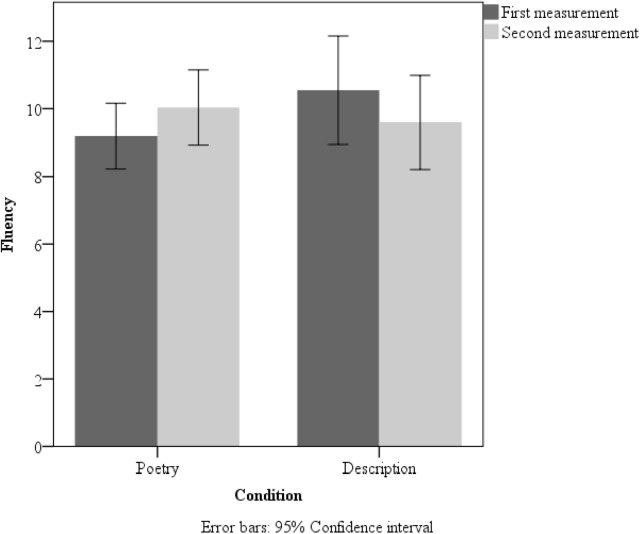
Mean fluency scores in the poetry-reading group and description-reading group in the first and second measurement in Study 1. Error bars: 95% Confidence interval.

A 2 × 2 (measurement × group) repeated measures ANOVA for fluency revealed an interaction [*F*(1,105) = 12.12, *p* < 0.001, η^2^ = 0.1], but no main effects. Pairwise comparisons showed a significant improvement in fluency scores on the second measurement compared to the first in the poetry group [*t*(56) = 2.57, *p* = 0.013; Cohen’s *d* = 0.35]. Moreover, the control group differed in fluency across the measurements. Specifically, participants in this group demonstrated significantly lower scores in the second measurement than in the first [*t*(52) = 2.44, *p* = 0.018; Cohen’s *d* = 0.35]. Extended data are shown in **Figure 1**.

**FIGURE 2:**
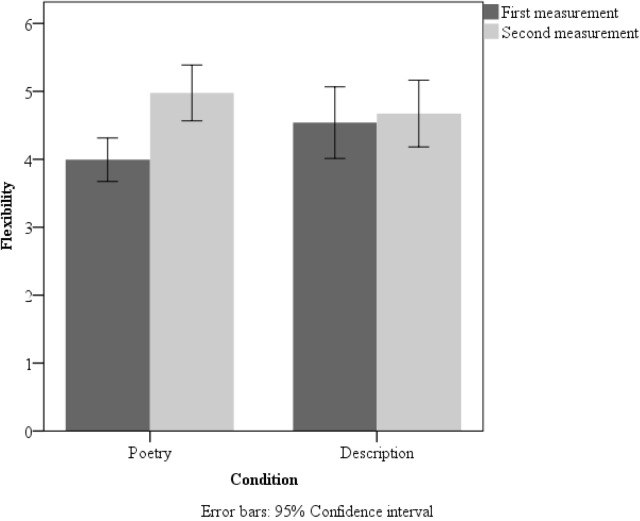
Mean flexibility scores in the poetry-reading group and description-reading group in the first and second measurement in Study 1. Error bars: 95% Confidence interval.

A 2 × 2 (measurement × group) repeated measures ANOVA for flexibility also revealed an interaction [*F*(1,105) = 10.15, *p* < 0.01, η^2^ = 0.09]. Further, a main effect of measurement was observed [*F*(1,105) = 17.52, *p* < 0.001, η^2^ = 0.14]. The second picture of the DT task led to more flexible answers (*M* = 4.83, *SD* = 1.63) than did the first one (*M* = 4.25, *SD* = 1.56). Two-tailed, paired *t*-tests for two measurements in the poetry group yielded significant differences [*t*(56) = 5.47, *p* = 0.001; Cohen’s *d* = 0.75]. Extended data are presented in **Figure 2**.

**FIGURE 3:**
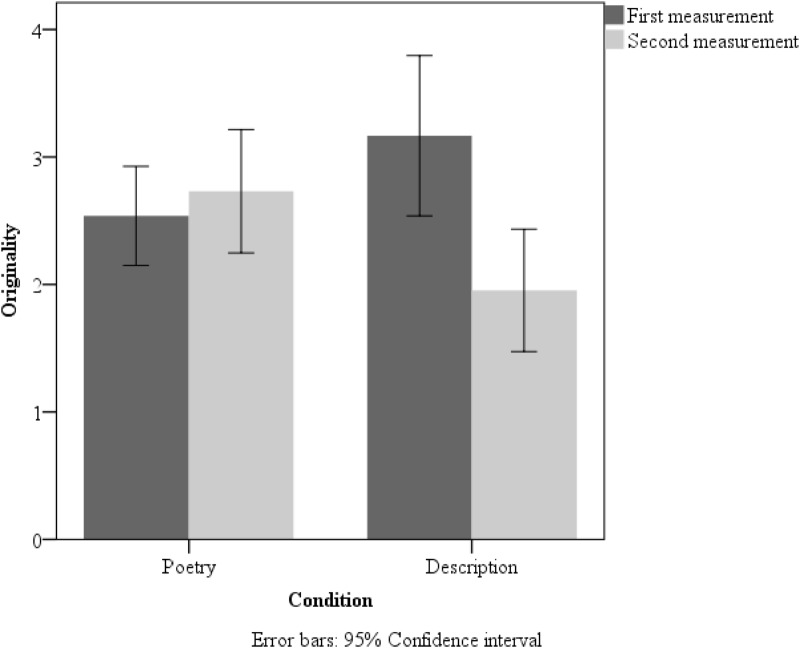
Mean originality scores in the poetry-reading group and description-reading group in the first and second measurement in Study 1. Error bars: 95% Confidence interval.

A 2 × 2 (measurement × group) repeated measures ANOVA for originality also revealed an interaction [*F*(1,105) = 23.03, *p* = 0.01, η^2^ = 0.18]. Additionally, a main effect of measurement was observed [*F*(1,105) = 12.12, *p* < 0.01, η^2^ = 0.11]. The first picture in the creativity test triggered more original answers (*M* = 2.85, *SD* = 1.18) than did the second (*M* = 2.34, *SD* = 1.71). Two-tailed paired *t*-tests yielded significant differences between the first and the second measurement only in the description group [*t*(50) = 5.09, *p* < 0.001; Cohen’s *d* = 0.75]. Extended data are shown in **Figure 3**.

To verify how individual differences in poetic interests are connected to DT, we also performed a linear regression analysis predicting DT on the first measurement (before the manipulation). As expected, flexibility was predicted by the level of poetic interests, *F*(1,56) = 3.29, *p* = 0.075, *b* = 0.24 (a near-significant trend). However, fluency and originality were not predicted by level of poetic interests. Further, no significant predictions were observed for the second measurement of creativity.

### Discussion

Results of the experiment support our hypotheses to a large extent, however, there are some issues that remain to be elucidated. Reading of poetry improved two creativity indicators (fluency and flexibility), while reading of the control (descriptive) text caused a decline in fluency and originality. Although these results are interesting, the question of why reading poetry does not improve originality remains. It is possible that reading this type of poetic narration introduces insufficient changes to the semantic network, so that individuals were unable to improve in the only indicator of product quality (i.e., originality). Additionally, flexibility did not decrease as a result of reading instructions. Likely because the cooker is compared with similar devices, which requires looking at it from different perspectives. Moreover, frequent contact with poetry predicted flexibility. These results suggest that the reception of narrative and open poetry broadens activation of the semantic network and allows for flexible switching between remote categories; however, it is not connected with the creation of very original solutions.

The chosen poem combines both abstract and concrete concepts. The abstract ones (e.g., *obvious, understanding*) are explained in concrete or imaginative terms (e.g., *valley, tree*), which facilitate a distinct view of reality (Kirsch and Guthrie, 1984). Contact with this kind of poetry can diversify experience, which can lead to increased flexibility (Ritter et al., 2012). Hence, poetry reception may result in diverse idea generation. Flexibility is the ability to use various categories beyond the boundaries of their literal meaning. Many researchers agree that reception of poetry inhibits automatic associations, thereby producing ideas without value (Kirsch and Guthrie, 1984; Halonen, 1995). Creative thinking is often connected with breaking typical patterns of thinking and seeing the world in another way (Amabile, 1996), which relates to intellectual risk-taking (Nickerson, 1999).

The lack of change in originality scores may be related to the character of the poem. *Utopia* is rather calm, balanced, and narrative. As such, it may be able to weaken resistance to seeing things from another point of view (flexibility). In contrast, reception of such a poem may inhibit original idea production until the whole of the poem is understood. Therefore, the reception of this type of poetry may have a buffering effect on intrinsically motivated original ideas. The purification of the dominant influence of the author’s unique perspective is possible in more emotional and cathartic poetry. Thus, increased originality may be more visible after reception of cathartic metaphoric poems, which presents the extraordinary experience of a poet.

Finally, showing that the level of poetic interest predicts flexibility (measured prior to manipulation) is in line with previous research; specifically, that long-term contact with poetry is associated with creative problem solving (McGovern and Hogshead, 1990). As Sternberg and Lubart (1999) claim, people’s interest in poetry can increase creative potential understood as seeing problems in unique ways.

Study 1 showed the positive impact of narrative poetry on DT. Subsequently, Study 2 utilized conventional poetry, with the hypothesis that reception of this type of poetry would not enhance creativity. We wanted also reveal why individuals demonstrate spontaneous contact with poetry, which may be essential for receiving this kind of art, and thus increased performance on tasks requiring DT ability. These elements were empathy (i.e., the tendency to become immersed in the poetry content; Davis, 1983), and need for cognition (NFC; construed as willingness to interact with the cognitively demanding text of a poem; Cacioppo and Petty, 1982). Poems can be challenging cognitive tasks. As such, understanding a poem requires the creation of complex meaning from specific words and exploration of multifaceted ideas (Csikszentmihalyi, 1996).

We predicted that the variables listed above would be crucial for initial DT levels (i.e., baseline, recorded during the first DT test); but that these individual difference effects would disappear after the manipulation. We also predicted that reception of conventional poetry (and the control text) would lead to a poorer performance on the DT task after its reception.

## Study 2

### Methods

#### Participants

Participants were recruited from high-school classes. All participants resided in Poland. A total of 131 participants completed the study (*M* age = 16.36; *SD* = 0.71; 84 female). Students from this pool were randomly assigned to one of two groups. Upon entering the lab, participants were given a consent form and a brief explanation of the study procedures. The study was conducted in a group setting, with the number of participants ranging from 10 to 15. Participants provided written, informed consent, and were free to withdraw from the research at any time without giving reason or justification for withdrawing and received course credit as compensation. Minors participated in research with written parental consent. Participants received course credit for participation, and their participation was anonymous. The study was approved by a local ethics committee (clearance number: WKE/S 15/VI/1).

### Materials

#### DT Measurement

DT measurement protocols for this study were identical to those used in Study 1.

#### Gustafson’s Poem

Lars Gustafson’s poetry is philosophical; descriptive; and uses well-known metaphors of “life as a machine,” which was very popular in the 20th century. We used the Polish version of Gustafsson (2013) poem, *Silence of The World before Bach*, which, in a very descriptive way, presents a biography of Bach and the changes in the world connected with his music/art works. It uses commonplace metaphors, which describe the world in well-known ways (e.g., “*Soprano never in helpless love twined round the gentler movements of the flute*”), making it an excellent example of conventional poetry. The chosen poem does not rhyme and is emotionally stable, which was confirmed by three judges, in a manner similar to Study 1.

#### Gustafson’s Poem Description

For a control text, we created a description of the poem’s content. It approximated the word count of the poem and did not contain any metaphors.

#### Contact With Poetry Scale

This scale was an extended version of the task created for Study 1, which measures passion for poetry, as well as frequency of poetry reading and taking part in poetic meetings (e.g., “*I am passionate about poetry,”* “*In my free time I very often read poems,”* and *“Poetry is incredibly difficult for me”*). Participants answered the eight items on a 5-point scale from 1 = strongly disagree to 5 = strongly agree. The reliability of the tool, as measured by internal consistency, was satisfactory (Cronbach’s α = 0.853).

#### The Rational Experiential Inventory—NFC (Reflective) Scale

We used the Polish version of the Rational Experiential Inventory (REI; Epstein et al., 1996; Shiloh et al., 2002). This tool consists of two dimensions: an analytical-rational style of thinking and an intuitive-experimental style of thinking. The REI was devised based on the Myers-Briggs Type Indicator (Briggs and Myers, 1976) and the NFC scale (Cacioppo and Petty, 1982), which defines the type of motivation described by the authors as the need for knowledge cognition. The NFC scale was used to build a rational (reflective) REI scale, opposite of the intuition scale. The most important element of this measure for the current study was the NFC scale. The REI is a 40-item Likert scale with response options ranging from 1 (*strongly disagree*) to 5 (*strongly agree*) The reliability of this tool, as measured by internal consistency, was satisfactory (Cronbach’s α for whole REI = 0.821, α for the NFC scale = 0.743).

#### Interpersonal Reactivity Index (IRI)—Fantasy Scale

The IRI is a questionnaire addressing empathy. It consists of four scales: Perspective Taking, Fantasy, Empathic Concern, and Personal Distress. In the current study, the Fantasy scale was used. This scale measures the tendency to imaginatively transpose oneself into fictional situations, as well as into the feelings and actions of fictitious characters in books, movies, and plays. This scale consists of 7 items (e.g., “*I really get involved with the feelings of the characters in a novel*,” “*I am usually objective when I watch a movie or play, and I do not often get completely caught up in it*”). The IRI involves a 5-point response option scale ranging from 1 (*strongly disagree*) to 5 (*strongly agree*). The reliability of the Fantasy Scale, as expressed by Cronbach’s α, was 0.682.

### Procedure

Participants first completed the baseline creativity test. Then, participants were randomized into one of two groups; (a) the experimental group that read the poem, and (b) the control group that read the description of its content. Participants read his/her respective documents twice. After the second reading, participants completed the second creativity test and completed the questionnaires listed above, using pen-and-paper procedures. The order of the creativity tests was counterbalanced across participants. After completing the scale, participants were debriefed and thanked for their participation. We also collected postal addresses from participants interested in the results.

Data were analyzed using SPSS 24 (IBM, Armonk, NY, United States). Two participants were excluded from analyses due to lack some data. A significance level of *p* < 0.05 was adopted for all tests.

### Results

All three DT indicators were scored by five independent raters. Kendall’s *W* = 0.9 for fluency in both measurements; *W* = 0.78 and 0.72 for flexibility in the first and the second measurement, respectively; and *W* = 0.7 for originality in both measurements. All indicators were analyzed separately by means of three repeated-measures ANOVAs with effect of measurement (first vs. second) as the within-subjects factor and group (poetry vs. description) as the between-subjects factor.

**FIGURE 4:**
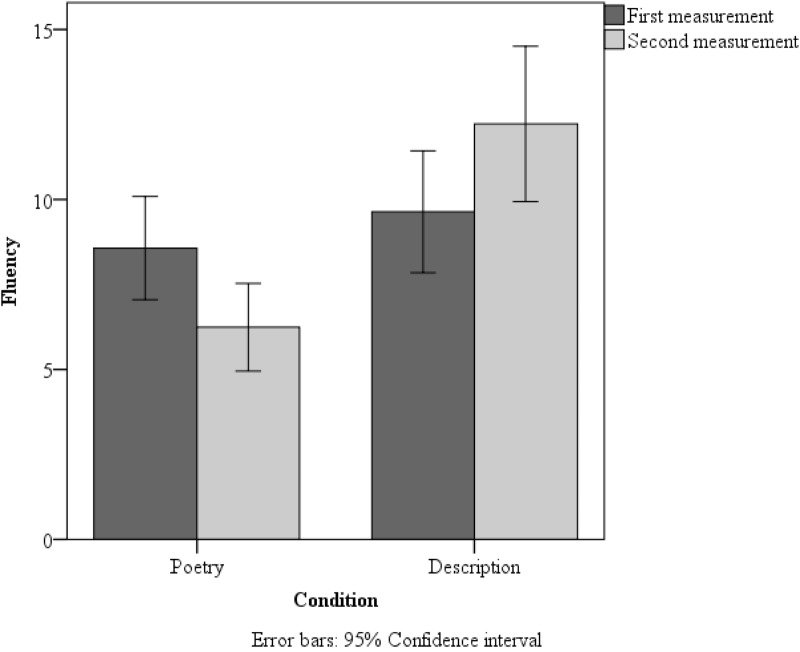
Mean fluency scores in the poetry-reading group and description-reading group in the first and second measurement in Study 2. Error bars: 95% Confidence interval.

A 2 × 2 (measurement × group) repeated measures ANOVA conducted for fluency revealed an interaction [*F*(1,127) = 11.56, *p* = 0.01, η^2^ = 0.08]. Moreover, we found a main effect of Group [*F*(1,127) = 12.35, *p* = 0.001, η^2^ = 0.09]. The poem made people less fluent (*M* = 7.41, *SD* = 0.71) than did the description (*M* = 10.93, *SD* = 0.72). Pairwise comparisons showed that, in the second measurement, the poetry group’s fluency was significantly lower than the fluency of the description group [*t*(127) = 4.61, *p* = 0.001; Cohen’s *d* = 0.84]. Two-tailed paired *t*-tests showed that the poetry group demonstrated a significant decrease in scores on the second measurement compared to the first measurement [*t*(65) = 2.52, *p* = 0.014; Cohen’s *d* = 0.31]. Furthermore, the description group demonstrated better scores on the second measurement than on the first [*t*(62) = 2.31, *p* = 0.024; Cohen’s *d* = 0.29]. Extended data are shown in **Figure 4**.

**FIGURE 5:**
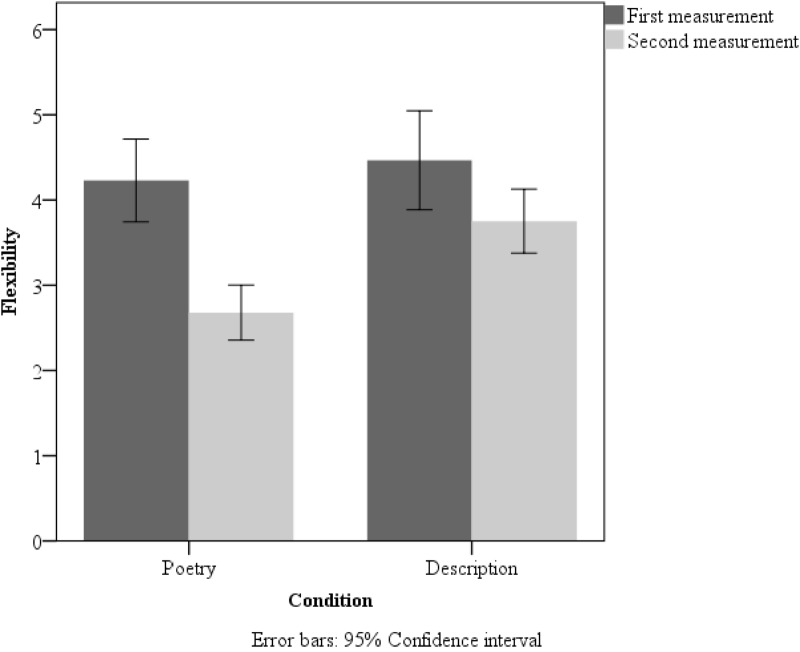
Mean flexibility scores in the poetry-reading group and description-reading group in the first and second measurement in Study 2. Error bars: 95% Confidence interval.

A 2 × 2 (measurement × group) repeated measures ANOVA for flexibility also revealed an interaction [*F*(1,127) = 3.92, *p* = 0.05, *η*^2^ = 0.03]. Additionally, we found a main effect of group [*F*(1,127) = 28.68, *p* < 0.001, *η*^2^ = 0.18]. The description triggered more flexible answers (*M* = 4.11, *SD* = 0.17) than did the poem (*M* = 3.45, *SD* = 0.17). We also found differences between the first and second measurement of flexibility in both the poetry [*t*(65) = 5.64; *p* = 0.001; Cohen’s *d* = 0.71] and description groups [*t*(62) = 2.21, *p* = 0.031; Cohen’s *d* = 0.29]. Two-tailed paired *t*-tests showed that flexibility of both groups dropped in the second measurement when we compared its level with the first measurement. Furthermore, we found differences between the poetry and the description groups in the second measurement [*t*(127) = 4.34, *p* = 0.001; Cohen’s *d* = 0.59]. Two *t*-tests showed that poetry reception resulted in lower flexibility scores than description reception in the second measurement. Extended data are presented in **Figure 5**.

A 2 × 2 (measurement × group) repeated measures ANOVA for originality yielded not significant interactions or main effects.

Next, we conducted linear regression analyses to determine whether the mean frequency of contact with poetry, fantasy (empathy factor), and/or NFC predicted DT scores in the baseline measurement. Analyses showed that frequent contact with poetry positively predicted all parameters of DT [fluency, *F*(1,127) = 21.49, *p* < 0.001, *R^2^* = 0.15, *b* = 0.38; flexibility, *F*(1,127) = 23.73, *p* < 0.001, *R^2^* = 0,16, *b* = 0.39; and originality, *F*(1,127) = 17.94, *p* < 0.001, *R^2^* = 0,13, *b* = 0.35]. Further regression analyses yielded no significant associations between DT and fantasy, or DT and NFC.

We tried to explain the observed behavior—contact with poetry—in psychological terms. To elucidate the impacts of personality predictors on contact with poetry, we performed a single multiple regression analysis. The dependent variable was frequency of contact with poetry and the independent variables were fantasy and NFC. Results showed that the two-variables model was significant: *F*(2,127) = 10.67, *p* < 0.001, *R^2^* = 0.15. Fantasy was a slightly stronger predictor of contact with poetry/passion (*b* = 0.26) than was NFC (*b* = 0.25). As predicted, we found no significant effects regarding these variables in the second measurement.

### Discussion

We found that contact with conventional, biographical poetry led to decreased indicators of DT. We also observed that people who received this type of poetry demonstrated less fluent and flexible thinking compared with those that read a description of the same information. These results provide support for our hypothesis that idea generation is less likely after reception of narrative-conventional poetry, and that people are less creative after reading this kind of text, when compared to reading a neutral text.

Kovecses (2010) stated that a large body of poetry is constructed in a very conventional way (i.e., based on conceptual, conventional metaphors that are often used in everyday language). Such conventional metaphors (e.g., life is a journey; death is dark), as a part of our cognitive system, allow us to adapt to reality, but do not necessarily stimulate creativity (Lakoff and Turner, 1989). “The idea that metaphor constrains creativity might seem contrary to the widely held belief the metaphor somehow liberates the mind to engage in divergent thinking” (Gibbs, 1994, p. 7). Poets create novel, non-conventional poems through cognitive transformations: elaboration, extension, questioning, and combining (Lakoff and Turner, 1989). Therefore, it seems that the biographical, closed, and conventional poetry is also insufficient to stimulate creativity.

Our research confirms that contact with poetry, understood as long-term individual interest (not one-time contact), is associated with readers’ creativity. Accordingly, the results showed that frequent contact with poetry could be explained by individual differences, specifically increased ability to become absorbed in the feelings of characters in a novel, as well as a stronger NFC. We can conclude that the features of the text, as well as the ability to actively perceive the poem, are key factors for appropriate poem reception. Noy and Noy-Sharav (2013) argue that the emotional message of art is always individually perceived. Silvia (2005), who refers to the appraisal theory of aesthetic emotions, claims that the evaluation of art, and not art itself, arouses emotions. Understanding of a poem requires the ability to actively follow and immerse oneself in the poetry content, which is an essential dimension of empathy (Davis, 1983). Experience suggests that absorption and poetry-elicited empathy should impact positively on the aesthetic evaluation of a poem (Garrido and Schubert, 2011; Taruffi and Koelsch, 2014).

Furthermore, curiosity is a key component of emotional motivation (Hoffman, 2006; Silvia, 2005). The recipient should be motivated to comprehend the cognitively demanding content of the poem, which is a determinant of NFC (i.e., an individual’s tendency to engage in, and enjoy, effortful cognitive endeavors; Cacioppo and Petty, 1982). In general, we conclude that poetry reception favors pro-creativity states only under certain conditions, and that these conditions should be investigated in future studies.

## General Discussion

Poets describe their emotions and observations, in the form of metaphorical statements, in an effort to better convey their vision of the world to the reader. In two studies, which were conducted using a test/re-test design, we controlled for the impact of two different types of poems, from two renowned artists, to determine what, if any, impact the reception of poetry has on idea generation. Szymborska’s narration is intellectually intriguing, with a surprising conclusion. Conversely, Gustafson’s narration is a poetic description of the music of a master. The first poet uses open metaphors, while the second conventional ones. We expected, and confirmed to a large extent, that perceiving novel metaphors, based on remote associations (i.e., open metaphors) would result in more creative responses to a problem, whereas reception of well-known metaphors, which reinforce the world view shared by the community (i.e., closed metaphors) would lead to less creative ideas. Even one-time contact with narrative, open poetry improved some aspects of DT. However, we did not observe changes in originality, which is the key indicator of DT efficiency. We attributed this effect to the author’s reasoning, aimed at one, surprising punch line.

Despite limitations in the selection of material, we conclude that poetry could be a useful tool for manipulating DT. Specifically, the results of the current studies suggest that poetry improves creativity if it contains open metaphors. However, reading conventional poetry may actually decrease idea generation. It is likely that the selection of poetic and control texts will remain an open problem for future studies on this topic.

We also accounted in these studies for individual differences that are critical for poetry reception. Frequent contact with poetry is associated with a slightly higher level of DT (compared to a lack of involvement in poetry) and could be explained by higher need for cognition (curiosity) and ability to empathize with poetry content.

### Limitations and Future Directions

Although many of our hypotheses about the varied impact of poetry on generating ideas have been confirmed, it became clear that the simple division of metaphors into novel/open and well-known is not enough of a manipulation to affect DT. The narrative structure of the poem introduced limitations to the free and original interpretation of even the most distant, metaphorical associations. Therefore, future studies will seek pro-creative poetry in less structured and more emotional forms of poetic expression, specifically with the development of emotional themes that increase uncertainty and stimulate the reader’s imagination (Kozielecki, 2007).

While we showed that the impact of poetry reading on creative thinking depends on the type of poetry, future studies should manipulate the type of poetry utilized in a single study. Specifically, there are more types of poetry (aside from non-conventional and conventional) that could impact the reader in diverse ways that we did not explore. According to Heiden (2014), a fictionalized, narrative text can either address one’s understanding of life and a specific challenge found within the individual’s personal story (reference to “I”), or be an interpretation of events in the form of a story in general (referenced as “life at large”). Poetry that focuses on feelings, and disregards coherent narration, can be referred to as “cathartic poetry” (omitted in this research). The aim of cathartic poems is not to bring meaning closer, but rather to evoke the reader’s emotions. This type of poetry is an open task for readers, because everybody can comprehend it according to his or her own experience and understanding. It can support creativity more than narrative poetry used in the Study 1. Thus, it would be desirable to use narrative, cathartic, and conventional poems in one experimental model.

The current studies showed no increase in originality following poetry exposure. Therefore, it is important to conduct future studies to determine what kind of poetry, as well as what kind of cognitive abilities are necessary to achieve an increase in originality, which is the primary metric in DT.

It is also possible that the effects we observed could be due to the specific poems chosen, rather than the content relating to metaphor styles. This issue can be addressed only by choosing several wide-ranging poems, which differ in terms of both metaphorization style and structure. In addition to the well-structured poetry that we used in the current studies, we will choose poems in future research that are emotional and uncertain.

It is important to note that the control texts used in both of our experiments were not rated by the same judges who rated the poems in terms of affectivity and comprehensibility. Thus, we did not control the same possible factors that were neutralized by selecting and rating poems. Future studies should seek to ensure that all pieces used (both poetry and control) are rated. Additionally, the description of the poem’s content that was used as control text in the second study expresses a similar meaning to the poem, but without the use of metaphors. Without rating the content of both texts (poetry and its description), however, we cannot infer their similarity. To address this, a diverse range of texts included in the final collection should be rated by judges in the same manner as poems, both for affectivity and comprehensibility. In this way, the collection would result in several poems, restricted to the best examples of the three different metaphor styles (i.e., narrative, conventional, and cathartic). Further, the personality determinants of poetry receiving in judges and the receivers should be also be controlled.

In the current studies, creativity was more related to general problem solving than production of creative works (e.g., poetry, fictional stories). In future studies, we intend to check the influence of specific types of poetry reading on creating one’s own poems or prose samples. Future research should also explore the underlying mechanism behind how poetry influences creativity. Considering factors like emotions that are a consequence of contact with a poem, as well as individual differences in NFC and empathy, would allow us to construct a model to better describe the impact of poetry on the human mind. Furthermore, we failed to target specific audiences with specific types of poetry, which future studies should attempt. Finally, since the sample comprised high school students it would be difficult to extrapolate the results to a wider population.

## Data Availability Statement

Datasets are available upon request. The raw data supporting the conclusions of this manuscript will be made available by the authors, without undue reservation, to any qualified researcher.

## Ethics Statement

The study was reviewed and approved by the Ethics Council of SWPS University of Social Sciences and Humanities, Faculty in Sopot, Poland. Written informed consent was obtained from all participants and from the parents of all minors.

## Author Contributions

MO and AK equally contributed to the study concept and design. Additionally, MO collected the data, developed the line of argumentation, performed the data analyses, and developed a poetry classification. MO and AK approved the final version of the manuscript for submission.

## Conflict of Interest Statement

The authors declare that the research was conducted in the absence of any commercial or financial relationships that could be construed as a potential conflict of interest. The reviewer PC and handling Editor declared their shared affiliation at time of review.
